# “The Ultimate Decision Is Yours”: Exploring Patients’ Attitudes about the Overuse of Medical Interventions

**DOI:** 10.1371/journal.pone.0052552

**Published:** 2012-12-26

**Authors:** David Schleifer, David J. Rothman

**Affiliations:** 1 Center on Medicine as a Profession, Columbia College of Physicians & Surgeons, New York, New York, United States of America; 2 Center on Medicine as a Profession, Columbia College of Physicians & Surgeons, New York, New York, United States of America; The University of New South Wales, Australia

## Abstract

Previous research has found that American patients strongly believe that more testing and more treatment lead to better outcomes and, to a lesser extent, that newer treatments are more effective. We conducted five focus groups with privately insured, healthy, middle-aged Americans (n = 43) to explore these apparent preferences. Contrary to previous research, an unexpected distinction emerged. Participants placed enormous value on testing and screening, reacting with hostility to guidelines recommending less of either. However, they were suspicious of overmedication. The wariness of pharmaceuticals and enthusiasm for testing and screening both appear to reflect participants’ efforts to take responsibility for their health. But recommendations to test and screen less conflicted with their active, engaged, information-seeking roles. Nonetheless, given patients’ concerns about overuse of pharmaceuticals, we maintain that they can learn to understand the connections between over-testing and over-treatment, and can actively choose to do less. We close with suggestions about how treatment guidelines can better communicate these connections to patients. Our findings cannot necessarily be generalized beyond privately-insured, healthy, middle-aged Americans. But because we found that, among these individuals, attitudes towards pharmaceuticals differ from attitudes towards testing and screening, we maintain that future research should also distinguish among and compare attitudes towards different types of medical interventions.

## Introduction

The overuse of medical interventions, including pharmaceuticals, testing, and screening, has come under scrutiny for its economic costs and potential to harm patients [1]–[5]. In the United States, unnecessary services cost private and public payers $210 billion in 2009, approximately 30% of all healthcare costs [6]. In Medicare, the US program that provides health insurance for all Americans over age 65, the number of diagnostic tests per beneficiary grew approximately 85% from 2002 to 2012 [7]. Drivers of overuse in the United States include fee-for-service payment structures, physicians’ fear of malpractice lawsuits, industry marketing of pharmaceuticals and devices to medical professionals, and physicians’ culture of thoroughness [8]. To address overuse, the American Board of Internal Medicine (ABIM) Foundation and a coalition of professional medical associations have begun to engage physicians in efforts to “choose wisely” among treatments and screening and testing procedures [9].

However, physicians are not solely responsible for the types and volume of clinical care that patients receive. Two generations ago, patients were expected to defer to physicians’ authority and accept the passivity inherent in what Talcott Parsons called the sick role [10]. Patients were meant to seek technically competent help and follow physicians’ orders. But patients’ and physicians’ roles have changed. Starting in the 1960s, bioethical, legal, and administrative principles promoted patient autonomy and circumscribed physicians’ discretion [11], [12]. The trend toward patient autonomy accelerated in the 1970s and 1980s, in part because of activism by feminists and people with AIDS who were determined to control their medical care [13]. Patient access to medical information through the internet has reduced their dependence on physicians for medical information [14], [15]. Although patient choice remains constrained by physicians, payers, regulators, and industry, patient participation has become an institutionalized part of the healthcare system [16].

Given patients’ participation in decision-making and care, their potential contributions to overuse have begun to draw attention. Carman et al found that patients strongly believe that more testing and more treatment lead to better outcomes and, to a lesser extent, that newer treatments are more effective [17]. We use the term “medical maximalism” to describe this reported preference for more medical intervention. Patients are especially enthusiastic about routine cancer screenings, regardless of false positives or limited effects on mortality [18]. Recent United States Preventive Services Task Force (USPSTF) recommendations to limit screening for breast and prostate cancer, for example, have provoked considerable public resistance [19]–[21]. Patients have also been found to be hostile to research-based treatment guidelines, which they believe could lead to rationing and “one size fits all” medicine [22].

Emanuel has attributed these maximalist attitudes to a general American enthusiasm for technology [8]. But Americans are not in fact enthusiastic about all technologies, nor do all Americans adopt technologies at the same rate [23]. Meanwhile, medical overuse is not solely an American problem [24]–[28]. In many economically more developed countries, the increased availability of screening and diagnostic technologies has made it possible for people to closely monitor their bodies for biomarkers associated with disease [29], [30]. However, specific features of the American healthcare system likely predispose patients and physicians towards maximalism. Third-party payers shield insured patients and their physicians from many of the costs of pharmaceuticals, testing, and screening. Pharmaceutical and device manufacturers now use direct-to-consumer advertising (DTC) to increase patient demand for and physician awareness of expensive new pharmaceuticals [31].

We sought to explore the logic underlying patients’ apparently maximalist attitudes, not to determine the quantitative distribution of their attitudes. We therefore conducted focus groups to better understand patient preferences for more pharmaceuticals, testing, and screening. We considered whether attitudes towards testing and screening differ from attitudes toward pharmaceutical use. We also sought to understand how problems of overuse and non-adherence could be related to patients’ efforts to take responsibility for their health.

## Methods

Participants (n = 43) were recruited from a database of individuals who had expressed interest in taking part in focus groups but who had not done so within the previous six months. Participants were selected based on self-reported answers to a screener developed by the authors (Appendix S1). Following Carman et al’s approach, we selected privately insured participants because previous research indicates that such individuals feel relatively empowered to make healthcare choices [17], [32]. We chose individuals who reported themselves and their immediate families as in “excellent” or “good” health because we assumed that people with terminal or chronic illnesses merit separate inquiry. We selected individuals in regular contact with the healthcare system, defined as having visited a physician 2–6 times and having filled 1–5 prescriptions for themselves in the past 12 months, excluding contraception. Individuals who reported more than one hospitalization in the past two years besides childbirth were excluded. Participants were middle-aged, employed full-time or part-time, spoke English fluently, had at least some college education and had household incomes above $50,000, the approximate 2011 median [33]. Anyone employed in healthcare was excluded, including medical professionals and employees of medical insurance companies, device manufacturers, or the pharmaceutical industry.

Recruiters asked potential participants to rate their agreement on a Likert scale with the statement: “More, more expensive, newer medical tests and procedures are usually better.” The screener also asked potential participants to rate on a Likert scale their satisfaction with the healthcare that they and their family receive. These questions were not used to qualify or disqualify potential participants, but served as prompts during the focus group discussions.

In March and April 2012, a trained moderator led five 60-minute focus groups using discussion guides developed by the authors (Appendix S2). Three groups were conducted in suburban locations – one in Tarrytown, NY and two in a suburban area of Atlanta, GA – and two in an urban location, Chicago, IL. However, we draw no conclusions about geographical variation. Focus group participants received $100 to compensate them for their time. The Institutional Review Board at Columbia University Medical Center approved the study and participants gave written informed consent (Appendix S3).

The focus group discussions were organized into three parts. First, the moderator briefly discussed participants’ degrees of satisfaction with their healthcare, including their attitudes about medical professionals, insurance companies, hospitals, and government entities roles in healthcare. Next, she initiated a longer discussion of maximalist and minimalist attitudes. This included discussing their responses to the screener question “More, more expensive, newer medical tests and procedures are usually better” and asking about instances in which they have felt over-treated and under- treated. Lastly, in order learn how participants respond to messages that challenge maximalist attitudes, the moderator solicited responses to popular media articles and editorials reporting recommendations to screen less, as well as a *Journal of the American Medical Association* editorial about the potential harms of cancer screening [34]–[43]. These conversational prompts mentioned recommendations to selectively limit prostate-specific antigen (PSA) testing, mammograms, pap smears, and annual physical exams; over-diagnosis of breast cancer and over-treatment of prostate cancer. We assumed that participants would be familiar with these procedures, thereby minimizing the need for explanation from the moderator.

The focus group discussions were observed by the authors. Audiotapes were professionally transcribed. The authors reviewed the transcripts independently and analyzed them jointly, in conjunction with the moderator. Ellipses in quotations from participants included below indicate that the authors have edited for expediency and clarity. Because focus group techniques are appropriate for assessing similarities among participants rather than differences between them, our analysis includes only themes that arose consistently across participants in all groups [44].

## Results

### Active Patients: “Determine What … Works for you.”

Focus group participants felt strongly that they should take responsibility for their health and be actively involved in their healthcare. As one individual stated, “I feel we need to take more personal responsibility for our own healthcare.” Several participants felt increasingly compelled to monitor and manage their health in middle age. As one noted, “You hit an age where … all of a sudden one person has one thing and one has another and you become a little more aware that you’re entering that period of your life.” Others discussed the need to stay healthy for the sake of their children. One participant noted, “You have to take care of yourself so you can take care of your family and they can grow and see you.”

This desire to be actively involved in their healthcare created a tension in participants’ descriptions of their relationships with physicians. As one individual maintained, “People need to be proactive. The doctor doesn’t always know everything.” Another agreed, “I don’t believe that the doctors are like God and you should just listen to whatever they have to say and believe them.” However, participants also reported a reluctance to directly question their physicians. One likened going to a doctor to going to the principal’s office, saying “You just don’t want to piss them off.” Another agreed that physicians “scold you and they reprimand you.” When the moderator asked whether participants asked their physicians about the necessity of tests or treatment, one participant said, “I think about it, but I don’t say it to him.” Another noted, “I think sometimes people are afraid to ask because you’re intimidated by your physician and I think these are powerful questions that not everybody always feels comfortable asking.”

Gathering information independently emerged as a way for participants to prepare for clinical encounters and to double-check or supplement information after appointments. As one participant said, “You can’t just go in there blind and whatever they tell you, you just do. You have to filter some of that information.” Several noted that physicians are pressed for time, and therefore as one said, “You have to be your own advocate and try to research.” According to yet another, “You’re going to have to determine what medicine works for you, what test works for you.” Participants reported going to multiple websites and “processing a multitude of information” because “you can’t trust one source.” They specifically cited WebMD, the Mayo Clinic and Consumer Reports as websites that could at least “take you down a chain of information,” but they did not recognize that some sites are commercially sponsored. Participants mentioned Dr. Oz’s website and television programs, although their faith in him was not absolute. According to one, “I listen to things [Dr. Oz] says and I try some things, but the ultimate decision is yours.” Some noted that online forums were useful ways to “get some real stories.” As one participant explained, “I have tried drugs before that have given me weird side-effects and my doctor will be like ‘No, I’ve never heard of it’ and then I’ll go on these forums and they’ll be like “Yeah, it gave me blurry vision.”

### The Appeal of Testing and Screening: “With the Tests, I Take Control of My Health”

Focus group participants enthusiastically embraced testing and screening as ways to actively and responsibly protect their health. Participants viewed these procedures as ways to diagnose diseases early and even to prevent their onset entirely. As one participant put it, “screening tests help to prevent things. So if you can detect things early that’s usually when you cure them.” One said, “I’d rather find something out earlier than later.” Another agreed, “If they catch you early, at least they can fix it.” Yet another maintained that testing could “prevent the big heart attack that’s going to kill you.” Several participants used terms like “early indication” and “early detection” or cited the ability of new technologies to “find things.” Testing and screening were also described more neutrally as “informative” and “feedback.” As one participant remarked, “It’s nice to get the pulse of your health.”

Participants described asking their physicians for more tests or wishing that testing were more frequent, “so that they can spot something.” One described himself as “a big believer in screen tests” and wondered why “people at a certain age” would not want to get as many tests as possible. Another told the group “I don’t want to risk missing cancer in my body and leaving my children without a mother.” One noted that compared to his healthcare, “I get more diagnostic information for my car when they do an oil change. We did a 100 point checklist on this thing. I wish they would do something like that.” Another said she takes as many tests as her insurance will cover: “I’m paying for it, I might as well get it.” Participants specifically raised and endorsed annual physical exams, electrocardiography, stress tests, colonoscopies, “blood workups,” PSA tests, and mammograms.

Focus group participants recognized that physicians earn income from testing and screening, which could motivate them to order unnecessary tests. One reminded the group that physicians have “invested in this million dollar machine and they have to pay it off.” Another stated simply, “it’s a business.” But while another participant acknowledged that physicians profit from testing, he reasoned it was nonetheless “better to get [tests] than not.” One participant expressed frustration with over-testing in emergency or acute settings, in which physicians are “just taking pot shots.” But in non-acute situations, he “would not mind taking more tests as a preventative measure … That gives me advanced notice.”

### Defending Testing and Screening: “You’re Stupid if you don’t do it.”

Because focus group participants viewed testing and screening as vital to safeguarding their health, they were hostile to messages that those procedures should be used less or could be harmful. They responded vigorously when the moderator read portions of articles reporting recommendations to test or screen less. For example, the headline of an *Atlanta Journal Constitution* article stated, “More healthcare doesn’t necessarily add up to better healthcare, especially if the ‘more’ comes in the form of procedures and tests” [43]. One participant responded, “That doesn’t make any sense. That’s like the stupidest thing I've ever heard.” Another agreed “That’s a dumb statement for dumb people to follow.” Others called such advice “propaganda” and “stupid as hell.” One stated “It just sounds like a moron would say something like that. It didn’t make any sense.” Several participants were struck by the contrast between messages to test less versus high-profile campaigns encouraging screening for breast cancer, heart disease, and prostate cancer. One person wondered, “What happens at 50 and they find you’ve got full blown [cancer] because you waited?” He insisted that testing and screening are “just common sense. You’re stupid if you don’t do it.” Another acknowledged that some tests are “only going to save these few lives, but what if one of those lives is your kid?”.

Participants accepted the anxiety associated with waiting for results. As one participant explained, given a choice of “a few weeks of stress as opposed to maybe finding lifesaving information, I’ll go through that any day.” Several related stories of false positives. A woman who had a false positive mammogram described her “peace of mind” when she eventually found out that she was free of breast cancer. The idea that the risk of false positives justified less testing struck one participant as “ridiculous” because “you wouldn’t know it’s a false positive until you go check it out further.” Another participant observed that “The problem is there are going to be people who feel psychologically more comfortable [getting screened] … no matter what statistics or who you get out there and what campaign you put out.”

The moderator asked participants about the types of information or messages that might prompt them to consider less testing or treating, but their responses were not sufficiently robust to include in our analysis. Instead, participants maintained that recommendations to test less represented insurance companies’ and government payers’ efforts to save money. One asked “Is this conventional wisdom driven by research or is it driven by somebody wanting to save money?” Another argued “I think it’s because they’re trying to plant the seed for government health care.” As one reasoned, “I think what it comes down to is they’re starting to employ cold, hard calculations and they’re looking at cost versus benefit.” Another noted ironically, “the whole premise was that if you see a doctor more often maybe you spot something that might be coming up and overall it would be cheaper for the industry if you can catch it at the early stages. We all bought into that, but we bought into it so much that they weren’t prepared for the cost.” Another similarly suspected that “all of a sudden they want to put the brakes on it because they don’t want to have to pay out.” Higher costs to the medical system did not justify less medical intervention for individuals. As several participants asked, “How can you put a price on my life?”.

### Overuse of Pharmaceuticals: “They Dish out too much Medication.”

While focus group participants viewed testing and screening as important ways to protect their health, they viewed pharmaceuticals very differently. Participants were concerned about both the overuse of pharmaceuticals and about side-effects. They had little trust in pharmaceutical companies, which one individual described as “out for your money.” Participants maintained that physicians were “being pressured by drug companies” to prescribe more and so “really try to push what they want you to do.” Several thought that physicians receive “kickbacks” for prescribing more. They were particularly sensitive to the overmedication of children. One explained, “Think about when we were kids. You got a cold and your mother said in a week it was gone. Now they’re sticking you full of pills.”

DTC advertising appeared to contribute to these attitudes. Many participants noted that the side-effects disclosures in commercials were disconcertingly long. One asked rhetorically “Do you really want to take something that could do all those things to you?” Another noted, “Hearing all those commercials and then they give you an hour’s worth of side-effects, I don’t want to take some of the stuff.” In addition to immediate side-effects, participants expressed concern about long-term negative consequences of pharmaceuticals. One worried about allowing her teenagers to take Accutane while another discussed a cousin who had breast cancer that she claimed was “directly related to getting estrogen.” Several noted that pharmaceutical companies were often subjects of lawsuits over long-term effects. One participant asked “How many times does a drug come on the market and people take it and then six months later they take it off the market and … and then six months later there’s an attorney on TV” soliciting for class-action lawsuit members?

Participants described refusing to take various medications, including statins, hormone replacement, and anti-osteoporosis drugs. Several mentioned allowing physicians to write prescriptions and then not filling them. One explained “I’ve taken the prescription, not filled it and done my own research, and changed my lifestyle, and the next time I went back, I didn’t need it, and I told her about it.” A woman remembered that her physician insisted she try hormone replacement therapy after her hysterectomy. “She wouldn’t stop bothering me, I just let her write the prescription and I just never take it.”

## Discussion

We found that participants placed enormous value on testing and screening. They reacted with hostility to messages recommending fewer procedures, using highly charged words like “stupid” and “dumb.” The rate of false positives was not a deterrent to testing. However, rather than wanting all medical interventions, participants were suspicious of pharmaceuticals. Several reported allowing physicians to write prescriptions that they did not fill or take. DTC advertisements actually contributed to their wariness. Although previous research has shown that DTC advertisements prompt requests for prescriptions, this wariness and reported non-adherence are consistent with other findings that patients choose not to follow pharmaceutical regimens in order to protect their health [45]–[48].

We acknowledge several limitations to our research. Qualitative methods do not indicate the proportion of individuals holding a given attitude. Nonetheless, the attitudes we found occurred consistently in all focus groups, indicating that they may reflect attitudes held more broadly by privately insured, middle-aged, financially stable Americans who are in contact with the healthcare system but are not seriously ill. We specifically sought to investigate attitudes among those types of people, and we cannot draw conclusions about other demographic groups or insurance cohorts. As discussed below, our findings raise timely questions about how other groups’ attitudes may differ. Furthermore, our findings are based on opinions stated by individuals in group settings. We did not observe clinical encounters or health-seeking activities. We assume that individuals’ stated opinions differ from their actual behaviors, but we cannot estimate the extent of those differences. In addition, focus groups provide only a snapshot of participants’ opinions. We cannot draw conclusions about how opinions change in response to political and economic events or in response to personal events such as illness, aging, and deaths of family members or financial setbacks. Finally, we did not collect data on race in our first focus group in Tarrytown NY and therefore cannot provide complete racial data for the sample. However, as stated in the methodology section above, focus groups are appropriate for assessing similarities among participants rather than differences between them. We therefore do not discuss race, gender, age, or geographic differences in our analysis and believe that the missing information about race does not significantly weaken our findings.

The limitations of our study suggest questions for future research, particularly about uninsured people’s attitudes about medical intervention. Uninsured and intermittently insured Americans use less healthcare than insured people do, regardless of income [49]–[51]. These lower rates of utilization are almost certainly associated with insurance status, rather than indicating any preference for less medical intervention. In fact, when previously uninsured people enroll in Medicare at age 65, they begin to use more healthcare than their previously insured peers [52]. Those higher rates of use by the newly insured likely reflect the poorer health status of uninsured people [53], [54]. But just as our focus group participants expressed opinions that reflected their experience as insured, financially secure participants in the healthcare system, future research should investigate how lack of insurance shapes others’ attitudes about overuse and appropriate care. Do uninsured people fear scarcity and therefore develop more maximalist attitudes about medical interventions? Or does lack of insurance make them more circumspect about the healthcare system in general and therefore less maximalist in their attitudes? These questions will become particularly important as millions of previously uninsured Americans enter the healthcare system given that the United States Supreme Court upheld the Patient Protection and Affordable Care Act and Barack Obama was reelected president in 2012.

Our discussions revealed how our participants’ attitudes towards pharmaceuticals differ from attitudes towards testing and screening. Future research on the insured, the uninsured, and the newly insured should identify and explore differences among attitudes towards other medical interventions. The analysis should include distinguishing between diagnostic testing and routine screening, between different modes of drug delivery, and between out-patient and in-patient procedures.

Our findings highlight the complexity of addressing the overuse of testing and screening. While patients are skeptical about pharmaceuticals, this perspective cannot necessarily be extended to testing and screening. Pharmaceuticals can give patients quick negative feedback in the form of side-effects, whereas the side-effects of testing and screening are not necessarily immediately apparent. Moreover, DTC pharmaceutical advertisements often list long rosters of side-effects, something not required for testing and screening procedures. Recommendations to test less effectively tell patients that they should have less information, which conflicts with their active, engaged, information-seeking roles. Despite the limited numbers of clinically relevant findings from screening procedures, no one wants to be among the tiny percentage of people who could have “caught it early.” The anxiety associated with false positives pales in comparison to the fear of uncertainty, disease and death. Furthermore, system-wide healthcare costs do not matter to individuals concerned about their own and their families’ health. In fact, discussions of costs may only reinforce suspicions that recommendations to test and screen less are designed to protect payers rather than patients.

The suspicion that guidelines are actually efforts by the federal government to cut costs suggests that the writing and distribution of guidelines needs fundamental alteration. First, both the professional medical associations that produce most guidelines and the USPSTF must clearly and continually communicate their independence from government payers. While that independence may be obvious to medical professionals and policymakers, it is not obvious to the public. Second, the guidelines writing process must engage healthy people who are at risk of excessive testing and screening. The Institute of Medicine’s (IOM) standards for improving guidelines recommend engaging disease-specific organizations in guideline development [55]. But disease-specific organizations can be expected to advocate for more screening, just as they advocate for more research funding [56]. Healthy people seeking preventive services, by contrast, are not represented by organizations and their interests are harder to define. Guidelines writing committees must therefore consider diseases-specific organizations’ biases and develop methods to include members of the public who are not patients. The USPSTF has invited public comments on its draft recommendations only since 2011. Whether those public engagement efforts include non-patients remains to be seen. Finally, the IOM does not discuss strategies for communicating guidelines beyond the medical profession. But given patients’ information-seeking activities, accessibly written guidelines should be published and publicized in the places online where people already seek information, rather than only in medical journals or on the USPSTF website.

Patients are not solely responsible for overuse. Even affluent, well-educated patients tend to see physicians as paternalistic and intimidating [57]. But we cannot rely solely on physicians to police overuse, just as we do not rely on them to police their own conflicts of interest [58]. Efforts to address overuse must involve professional medical associations, hospital systems, payers, and medical schools in modifying fee-for-service payment systems, enabling better coordination of care, and integrating lessons about overuse into training and continuing education. But the preferences of active patients nonetheless merit attention. Both the mistrust of pharmaceuticals and the enthusiasm for testing and screening reflect individuals’ efforts to take care of their health. The challenge is to engage patients in understanding the connection between over-testing and over-treatment, to see both as detrimental to their health, and to actively choose to do less.

## Supporting Information

Appendix S1 **Focus group screener.**
(DOCX)

Appendix S2 **Focus group discussion guide.**
(DOCX)

Appendix S3 **Consent form.**
(PDF)

Table S1 **Characteristics of focus group participants.**
(DOCX)

## References

[1] Grady D, Redberg RF (2010) Less Is More: How Less Health Care Can Result in Better Health. Archives of Internal Medicine 170: 749–750. 20458080 10.1001/archinternmed.2010.90

[2] Korenstein D, Falk R, Howell EA, Bishop T, Keyhani S (2012) Overuse of Health Care Services in the United States: An Understudied Problem. Archives of Internal Medicine 172: 171–178. 22271125 10.1001/archinternmed.2011.772

[3] Fisher ES (2003) Medical Care – Is More Always Better? New England Journal of Medicine 349: 1665–1667. 14573739 10.1056/NEJMe038149

[4] Ashton CM, Souchek J, Petersen NJ, Menke TJ, Collins TC, et al (2003) Hospital Use and Survival among Veterans Affairs Beneficiaries. New England Journal of Medicine 349: 1637–1646. 14573736 10.1056/NEJMsa003299

[5] Keyhani S, Falk R, Bishop T, Howell E, Korenstein D (2012) The Relationship Between Geographic Variations and Overuse of Healthcare Services: A Systematic Review. Medical Care 50: 257–261. 22329997 10.1097/MLR.0b013e3182422b0f

[6] Young PL, Olsen L, Medicine RoE-B, Medicine IO (2010) The Healthcare Imperative: Lowering Costs and Improving Outcomes: The National Academies Press. 21595114

[7] Hood VL, Weinberger SE (2012) High value, cost-conscious care: An international imperative. European Journal of Internal Medicine 23: 495–498. 22863424 10.1016/j.ejim.2012.03.006

[8] Emanuel EJ, Fuchs VR (2008) The Perfect Storm of Overutilization. JAMA: The Journal of the American Medical Association 299: 2789–2791. 18560006 10.1001/jama.299.23.2789

[9] ABIM Foundation (2012) Choosing Wisely. Philadelphia, PA: American Board of Internal Medicine.

[10] Parsons T (1951) Social Structure and Dynamic Process: The case of modern medical practice. The social system. Glencoe, IL: Free Press. 428–479.

[11] Rothman DJ (1991) Strangers at the bedside: A history of how law and bioethics transformed medical decision making. New York, NY: BasicBooks. xi, 303 p.

[12] Brody DS (1980) The Patient's Role in Clinical Decision-Making. Annals of Internal Medicine 93: 718–722. 7212484 10.7326/0003-4819-93-5-718

[13] Epstein S (2008) Patient Groups and Health Movements. In: Hackett EJ, Amsterdamska O, Lynch M, Wajcman J, editors. The Handbook of Science and Technology Studies. 3rd ed. Cambridge, MA: MIT Press. 499–539.

[14] Henwood F, Wyatt S, Hart A, Smith J (2003) ‘Ignorance is bliss sometimes’: Constraints on the emergence of the ‘informed patient’ in the changing landscapes of health information. Sociology of Health & Illness 25: 589–607. 12919447 10.1111/1467-9566.00360

[15] Broom A (2005) Virtually He@lthy: The Impact of Internet Use on Disease Experience and the Doctor-Patient Relationship. Qualitative Health Research. 15: 325–345. 10.1177/1049732304272916 15761103

[16] Institute of Medicine (2001) Crossing the quality chasm: A new health system for the 21st century. Washington, D.C.: National Academy Press. xx, 337 p. 25057539

[17] Carman KL, Maurer M, Yegian JM, Dardess P, McGee J, et al (2010) Evidence That Consumers Are Skeptical About Evidence-Based Health Care. Health Affairs 29: 1–7. 10.1377/hlthaff.2009.0296 20522522

[18] Schwartz LM, Woloshin S, Fowler J, Floyd J, Welch HG (2004) Enthusiasm for cancer screening in the United States. JAMA 291: 71–78. 14709578 10.1001/jama.291.1.71

[19] Barker KK, Galardi TR (2011) Dead by 50: Lay expertise and breast cancer screening. Social Science and Medicine 72: 1351–1358. 21440969 10.1016/j.socscimed.2011.02.024

[20] Davidson AS, Liao X, Magee BD (2011) Attitudes of women in their forties toward the 2009 USPSTF mammogram guidelines: a randomized trial on the effects of media exposure. American Journal of Obstetrics and Gynecology 205: 30.e31–30.e37. 22088897 10.1016/j.ajog.2011.04.005

[21] Arkes HR, Gaissmaier W (2012) Psychological Research and the Prostate-Cancer Screening Controversy. Psychological Science 23: 547–553. 22555966 10.1177/0956797612437428

[22] Gerber AS, Patashnik EM, Doherty D, Dowling C (2010) A National Survey Reveals Public Skepticism About Research-Based Treatment Guidelines. Health Affairs 29: 1882–1884. 20921489 10.1377/hlthaff.2010.0185

[23] Schepers J, Wetzels M (2007) A meta-analysis of the technology acceptance model: Investigating subjective norm and moderation effects. Information and Management 44: 90–103.

[24] James CD, Hanson K, Solon O, Whitty CJM, Peabody J (2011) Do doctors under-provide, over-provide or do both? Exploring the quality of medical treatment in the Philippines. International Journal for Quality in Health Care 23: 445–455. 21672923 10.1093/intqhc/mzr029 PMC3136200

[25] Shin SM, Kim MJ, Kim ES, Lee HW, Park CG, et al (2010) Medical Aid service overuse assessed by case managers in Korea. Journal of Advanced Nursing 66: 2257–2265. 20626489 10.1111/j.1365-2648.2010.05364.x

[26] Hemo B, Shamir-Shtein NH, Silverman BG, Tsamir J, Heymann AD, et al (2009) Can a Nationwide Media Campaign Affect Antibiotic Use? American Journal of Managed Care 15: 529–534. 19670956

[27] McIntyre MJ, Chapman Y, Francis K (2011) Hidden costs associated with the universal application of risk management in maternity care. Australian Health Review 35: 211–215. 21612736 10.1071/AH10919

[28] Long Q, Klemetti R, Wang Y, Tao F, Yan H, et al (2012) High caesarean section rate in rural China: Is it related to health insurance (New Co-operative Medical Scheme)? Social Science & Medicine 75: 733–737. 22595072 10.1016/j.socscimed.2012.03.054

[29] Armstrong D (1995) The rise of surveillance medicine. Sociology of Health & Illness 17: 393–404.

[30] Welch HG (2011) Overdiagnosed: Making people sick in the pursuit of health. Boston MA: Beacon Press.

[31] Donohue JM, Cevasco M, Rosenthal MB (2007) A Decade of Direct-to-Consumer Advertising of Prescription Drugs. New England Journal of Medicine 357: 673–681. 17699817 10.1056/NEJMsa070502

[32] Kaiser Family Foundation (2011) Kaiser Health Tracking Poll, August 2011. Menlo Park, CA: Kaiser Family Foundation.

[33] DeNavas-Walt C, Proctor BD, Smith JC (2012) Current Population Reports, P60–243, Income, Poverty, and Health Insurance Coverage in the United States: 2011. Washington DC: U.S. Census Bureau.

[34] Parker-Pope T (2012) New Pap Smear Guidelines Advise Less Frequent Tests. The New York Times. A21.

[35] Rabin RC (2012) Doctor Panels Recommend Fewer Tests For Patients. The New York Times. A.10.

[36] Woolf SH, Harris R (2012) The Harms of Screening. JAMA: The Journal of the American Medical Association 307: 565–566. 22318274 10.1001/jama.2012.100

[37] Chicago Tribune Editorial Board (2011) Better not to know?: For men, perplexing new advice on cancer detection. Chicago Tribune. 12–11.12.

[38] Chapman S (2011) When fighting cancer is folly. Chicago Tribune.

[39] Brawley O (2012) ‘Overdiagnosis’ of breast cancer may be higher than thought. CNN Health.

[40] Parker-Pope T (2012) The Annual Appointment Loses Some Relevance. The New York Times: D.5.

[41] Rosenbaum L (2012) How human nature drives up health costs. The Boston Globe. A.15.

[42] Woloshin S (2012) Endless Screenings Don’t Bring Everlasting Health. The New York Times. D.5.

[43] Tucker C (2009) Sometimes, healthcare “rationing” makes sense. Atlanta Journal Constitution.

[44] Morgan DL (1997) Focus groups as qualitative research. Thousand Oaks, CA: Sage Publications. viii, 80 p.

[45] USA Today, Kaiser Family Foundation, Harvard School of Public Health (2008) The Public on Prescription Drugs and Pharmaceutical Companies. Menlo Park, CA.

[46] McHorney CA, Spain CV (2011) Frequency of and reasons for medication non-fulfillment and non-persistence among American adults with chronic disease in 2008. Health Expectations 14: 307–320. 20860775 10.1111/j.1369-7625.2010.00619.x PMC5060587

[47] Donovan JL, Blake DR (1992) Patient non-compliance: Deviance or reasoned decision-making? Social Science & Medicine 34: 507–513. 1604357 10.1016/0277-9536(92)90206-6

[48] Horne R, Weinman J (1999) Patients’ beliefs about prescribed medicines and their role in adherence to treatment in chronic physical illness. Journal of Psychosomatic Research 47: 555–567. 10661603 10.1016/s0022-3999(99)00057-4

[49] Sudano JJ, Baker DW (2003) Intermittent Lack of Health Insurance Coverage and Use of Preventive Services. American Journal of Public Health 93: 130–137. 12511402 10.2105/ajph.93.1.130 PMC1447707

[50] Ayanian JZ, Weissman JS, Schneider EC, Ginsburg JA, Zaslavsky AM (2000) Unmet health needs of uninsured adults in the United States. JAMA: The Journal of the American Medical Association 284: 2061–2069. 11042754 10.1001/jama.284.16.2061

[51] Ross JS, Bradley EH, Busch SH (2006) Use of health care services by lower-income and higher-income uninsured adults. JAMA: The Journal of the American Medical Association 295: 2027–2036. 16670411 10.1001/jama.295.17.2027

[52] McWilliams JM, Meara E, Zaslavsky AM, Ayanian JZ (2007) Use of Health Services by Previously Uninsured Medicare Beneficiaries. New England Journal of Medicine 357: 143–153. 17625126 10.1056/NEJMsa067712

[53] Wilper AP, Woolhandler S, Lasser KE, McCormick D, Bor DH, et al (2008) A National Study of Chronic Disease Prevalence and Access to Care in Uninsured U.S. Adults. Annals of Internal Medicine 149: 170–176. 18678844 10.7326/0003-4819-149-3-200808050-00006

[54] Baker DW, Sudano JJ, Albert JM, Borawski EA, Dor A (2001) Lack of Health Insurance and Decline in Overall Health in Late Middle Age. New England Journal of Medicine 345: 1106–1112. 11596591 10.1056/NEJMsa002887

[55] Institute of Medicine Committee on Standards for Developing Trustworthy Clinical Practice Guidelines, Graham R, Mancher M, Dianne Miller Wolman, Greenfield S, et al. (2011) Clinical practice guidelines we can trust. Washington, DC: National Academies Press. 24983061

[56] Best RK (2012) Disease Politics and Medical Research Funding. American Sociological Review 77: 780–803.

[57] Frosch DL, May SG, Rendle KAS, Tietbohl C, Elwyn G (2012) Authoritarian Physicians And Patients’ Fear Of Being Labeled ‘Difficult’ Among Key Obstacles To Shared Decision Making. Health Affairs 31: 1030–1038. 22566443 10.1377/hlthaff.2011.0576

[58] Chimonas S, Patterson L, Raveis VH, Rothman DJ (2011) Managing Conflicts of Interest in Clinical Care: A National Survey of Policies at US Medical Schools. Academic Medicine 86: 293–299. 21248603 10.1097/ACM.0b013e3182087156

