# Supplementary material for: “The Ultimate Decision Is Yours”: Exploring Patients’ Attitudes about the Overuse of Medical Interventions
Source: PLoS One. 2012 Dec 26;7(12):e52552. doi: 10.1371/journal.pone.0052552 (PMC3530438; doi:10.1371/journal.pone.0052552)
Supplement: Appendix S1 — Focus group screener. (DOCX) [file pone.0052552.s001.docx]

Appendix A: Screener for CMAP Focus Groups

Prepared by the authors in conjunction with Karen Dubinsky, Marketing Insights

Hello, my name is _______ and I’m calling on behalf of Marketing Insights, a marketing research firm. We’re conducting research on people’s thoughts and opinions regarding healthcare. We’re inviting individuals who meet certain qualifying criteria to attend a focus group. Do you have time to answer a few questions?

We are not selling anything and if you qualify and we invite you to participate in the focus group you will not receive a sales call or be asked to buy anything. All information that we gather in the research, including the content of this survey will be kept confidential. If you are selected to participate in the focus group, you will be compensated for your time.

Would you like to continue with the survey to see if you qualify?

Aim to recruit a total of 10 respondents

- Male / female
- Ethnicity

1. Which of the following age categories do you fall within?

25-39 _________________Disqualify

40-50 _________________Qualify

51-60 _________________Qualify

61+ ___________________Disqualify

2a. What is your occupation? (Anyone who works in any area of the healthcare, medical insurance, medical equipment, pharmaceutical industry is disqualified.)

2b. Do you work in any of the following industries?

Insurance _____________Ask: What Kind?

…If medical insurance: Disqualify

Healthcare ___________Disqualify

Pharmaceutical _______Disqualify

2c. What is your work situation?

Full Time_______________ Qualify

Part Time_______________ Qualify

Retired_________________ Disqualify

Unemployed ______Disqualify

Homemaker _____________Qualify (Not More Than 2 Per Group)

3. Which of the following best describes your annual household income?

Under $25,000____________Disqualify

$26,000-$49,000____________Disqualify

$50,000-$99,000____________Qualify… Recruit A Mix

$100,000-$199,000__________Qualify… Recruit A Mix

$200,000 +________________ Qualify… Recruit A Mix

4. What is your family make-up?

Single ______________________Qualify, Not More Than 2 Per Grp

Married, No Children ________Qualify, Recruit A Mix

Married, Have Children ______Qualify, Recruit A Mix

5. Which of the following best describes your highest level of education?

High School __________________Disqualify

Some College ________________Qualify, Recruit A Mix

College ______________________Qualify, Recruit A Mix

Post-Graduate _______________Qualify

6. Do you have health insurance?

Yes _____________

Through employer or spouse’s employer? Qualify

No ______________Disqualify

7. How would you describe the current health situation of you and your immediate family (children, spouse, parents, siblings)?

Excellent Qualify------------------------------------------

Good Qualify-------------------------------------------

Fair Disqualify---------------------------------------

Poor Disqualify----------------------------------------

8a. How many times have you personally visited a physician this year (includes: psychiatrist, dentist, ophthalmologist)

- None _________________ Disqualify
- Once_________________ Disqualify
- 2-5 Times ______________Qualify
- 6 Or More Times _______Disqualify

8b. How many times have you been hospitalized in the past 2 years?

- None________________ Qualify
- Once________________ Qualify
- 2-3 Times _____________ Disqualify
- More Than 3 Times _______Disqualify

8c. How many prescriptions for yourself have you filled this year?

- None _______________Disqualify
- One ________________Qualify
- 2-4 _________________Qualify
- 5 Or More____________ Qualify

8d. How many prescriptions do you take on a regular basis?

- None____________ Disqualify
- One_____________ Qualify
- Two____________ Qualify
- 3-4 _____________ Disqualify
- 5 Or More______ Disqualify

9. Do you agree or disagree with this statement:

“More, more expensive, newer medical tests and procedures are usually better”

1. Totally agree__________
2. Somewhat agree_________
3. Neither agree nor disagree__________
4. Somewhat disagree____________
5. Totally disagree_____________

(This question is for information purposes only. It is not a qualifier for the groups.)

10. How satisfied are you today with the healthcare that you and your family receive?

Extremely satisfied________

Satisfied______________

Somewhat Satisfied _________

Somewhat Dissatisfied_______

Dissatisfied_____________

Extremely Dissatisfied_________

(This question is for information purposes only. It is not a qualifier for the groups.)

11. Why do you feel that way? (We want to make sure we get thoughtful, articulate respondents.)
