# Supplementary material for: “The Ultimate Decision Is Yours”: Exploring Patients’ Attitudes about the Overuse of Medical Interventions
Source: PLoS One. 2012 Dec 26;7(12):e52552. doi: 10.1371/journal.pone.0052552 (PMC3530438; doi:10.1371/journal.pone.0052552)
Supplement: Appendix S2 — Focus group discussion guide. (DOCX) [file pone.0052552.s002.docx]

Appendix B: Discussion Guide for CMAP Focus Groups

Prepared by the authors in conjunction with Karen Dubinsky, Marketing Insights

**Objectives**

Explore consumer attitudes and needs that reinforce the commitment to “more, newer, more expensive medicine is better medicine.”

**Who We’re Talking To**

Men and Women 40-60.

Have recent knowledge of/exposure to healthcare but no life threatening situations.

**I. Background/Introductions (10 min)**

Purpose of the groups: To discuss your thoughts and feelings about certain areas of healthcare.

**II. Exploring Attitude Context: Where Are They Coming From (20 min)**

1. How satisfied are you with you and your family’s current healthcare?

Rate on a scale of 1-5 (compare vs. screener) and discuss:

- What are you thinking when you hear the term healthcare? Probe: doctors, nurses, insurance companies, hospitals, and government.
- What are the things that make you feel satisfied about your healthcare?
- What’s good about your healthcare today?
- What are the things that make you feel dissatisfied?
- What’s bad about your healthcare today? Probe: insurance, doctors, nurses, insurance companies, hospitals, and government.
- What would make your healthcare ideal?

2. Do you have a primary care physician? How satisfied are you with your primary care physician or the doctor you see most often?

Rate on a scale of 1-5 and discuss:

- What words would you use to describe the relationship you have with your doctor?
- What do you like best about your doctor?
- What do you like least?
- When your doctor tells you that you need a test, what’s your first reaction?
- Do you discuss it, research it, get another opinion? Why/why not?
- Can you think of any other situation where you follow someone’s advice like that?

**III. Exploring “Less is More” and “More is More” Attitude (30)**

1. Discuss responses to screener questions: “More, more expensive, newer medical tests and procedures are usually better.”

- Tell me why you agreed/disagreed?
- Have you/your family had any specific experiences in this situation?
- Have you ever undergone a test/treatment that you felt was unnecessary, even though your doctor had recommended it?
- Have you ever asked for more diagnostics or treatment?
- Have you ever asked for less diagnostics or treatment?
- Have you ever worried that you were being over-treated?
- Have you ever worried that you were being under-treated?
- Have you ever discussed scientific evidence with your physician?
- Have you ever had an experience where you took a “less is more” approach to your health?
- If so, what led you to the conclusion that less is more?
- Are there circumstances in which you believe that less treatment care leads to better outcomes?
- Do you believe that there are risks as well as benefits to tests or screening?
- Discuss perceived risks and benefits

2. Can you think of other situations where you changed your mind or your behavior regarding your healthcare?

3. The newer the drugs, the better the health outcomes.

- Do you agree/disagree?
- Have you had any personal experiences in this area?
- Have you read anything about this? Where?
- Can you think of other situations where the newest isn’t necessarily the best?

4. Brand name drugs are generally more effective than generics.

- Do you agree/disagree?
- Did you always feel this way?
- What changed your mind?

5. Information:

- What if there was new information on this issue?
- What kind of information?
- What kind of sources might make you think differently about this?
- Can you think of what information would help you better assess these kinds of situations? Where would you like that information to come from?
- Where do you get the best information now?
- Discuss: use of websites, like WebMD; perceptions of these websites: are they commercial businesses? Who sponsors them?

**IV. Messaging: (30)**

Read headlines/show recent articles and discuss: How do you react to articles like this? How do they affect your thinking or behavior?

Where would the message have more weight for you?

Who should the message be from?

**V. Closing**

Any questions from the Team?

Any final thoughts from participants?

Do you think it’s important to communicate this kind of information to people?

Why/why not? What do you think is the biggest problem in doing this?

How would you explain the “less is more” idea to a physician? To a child? To a friend?
