# Supplementary material for: “The Ultimate Decision Is Yours”: Exploring Patients’ Attitudes about the Overuse of Medical Interventions
Source: PLoS One. 2012 Dec 26;7(12):e52552. doi: 10.1371/journal.pone.0052552 (PMC3530438; doi:10.1371/journal.pone.0052552)
Supplement: Appendix S3 — Consent form. (PDF) [file pone.0052552.s003.pdf]

Columbia University Consent Form

Consent Number: **CF-AAAK2713**
Participation Duration: 90 minutes
Anticipated Number of Subjects: 118

Contact

Contact	Title	Contact Type	Numbers
David Rothman	Professor	Principal Investigator	Telephone: 212-305-4096
Sarah Humphreys	Support Staff	Study Coordinator	Telephone: 212-342-5268

Research Purpose

The purpose of the study is to understand individuals' attitudes towards appropriate medical care. The results of the research will inform academic papers, as well as to help frame messages for public communication.

Information on Research

Study purpose

We are doing this research study to find out individuals' perspectives on appropriate and inappropriate healthcare. You are being asked to take part in this study because you are privately insured and have had some contact with the healthcare system in the past twelve months. About 118 people are expected to be enrolled in this study at four sites.

Invitation to Participate

The purpose of this form is to give you information to help you decide if you want to take part in a research study. This consent form includes information about: why the study is being done; the things that you will be asked to do if you are in the study; any known risks involved; any potential benefit; and options, other than taking part in this study, that you have. The principal investigator (the lead researcher for this project) David Rothman will discuss the study with you. If at any time you have questions about the study, please ask a member of the study team. Take all the time you need to decide whether you want to take part in this research study. The purpose of this research is described below in the 'What is Involved in This Study?' section of this consent form.

Procedures

Participation in the study involves discussing your viewpoints in a focus groups. Taking part in this study will last 90 minutes during one focus group session.

Audio recording

We are asking for you to allow us to audiotape you as part of the research study. The recordings will be used for analysis by the research team. The audio recording may include first names used during the focus group discussion. The recordings will be stored in a locked file cabinet with no link to your identity and will be destroyed after three years.

Risks

To the best of our knowledge, taking part in this study will not hurt you. A risk of taking part in this study is the possibility of a loss of confidentiality. Loss of confidentiality includes having your personal information shared with someone who is not on the study team and was not supposed to see or know about your information. The study team plans to protect your confidentiality. Their plans for keeping your information private are described in the 'confidentiality' section of this consent form.
If you give us information which suggests that your child or any other child is being abused, we are required by law to report that information to the Administration for Children's Services (ACS). Reporting this information may put you, your family, or others who are involved at risk of questioning and legal action by the authorities.
Telling us about your involvement in illegal activities involves the risk of criminal penalties and/or prosecution if your identity were to be revealed. In some cases, we may be required to report such information. If you give us information that you may hurt yourself or someone else, we must report this information to the authorities.
Although it is not a risk, taking part in this study involves the inconvenience of giving 90 minutes of your time in order to participate in the focus group, plus travel time to the focus group facility.

Benefits

You will not receive personal (direct) benefit from taking part in this research study. However, the information collected from this research may help others in the future.

Alternative Procedures

You may choose not to take part in this research study.

Confidentiality

Any information collected during this study that can identify you by name will be kept confidential. We will do everything we can to keep your data secure, however, complete confidentiality cannot be promised. Despite all of our efforts, unanticipated problems, such as a stolen computer may occur, although it is highly unlikely. Transcripts of your focus group will be assigned a code number, and separated from your name or any other information that could identify you. The research file that links your name to the code number will be kept in a locked file cabinet and only the investigator and study staff will have access to the file.
The following individuals and/or agencies will be able to look at and copy your research records:
- The investigator, study staff and other medical professionals who may be evaluating the study.
- Authorities from Columbia University and New York Presbyterian Hospital, including the Institutional Review Board ('IRB')
- The Office of Human Research Protections ('OHRP')

This study is not sponsored. No government regulatory agencies are involved in this study.

Compensation

You will receive $100 as a token of our appreciation for your time. The researchers will need to record your name and home address in order to pay you.

Additional Costs

There are no costs to you for taking part in this study.

Voluntary Participation

Participation in this study is voluntary. Refusal to participate will involve no penalty or loss of benefits to which you are otherwise entitled. You may discontinue participation at any time without penalty or loss of benefits to which you are otherwise entitled.

Additional Information

If you have any questions or concerns about the study or are hurt while taking part in this research study, you may contact David Rothman, djr5@columbia.edu, 212-305-4096 or Sarah Humphreys, sah2103@columbia.edu, 212-342-5268. You contact them by mail at: Center on Medicine as a Profession, Columbia College of Physicians & Surgeons, 630 W. 168th Street, Suite 1525, New York, NY 10032.

If you have any questions about your rights as a subject, you may contact: Institutional Review Board Columbia University Medical Center 722 West 168th Street, 4th Floor New York, NY 10032 Telephone: (212) 305-5883 or by email at irboffice@columbia.edu. An Institutional Review Board is a committee organized to protect the rights and welfare of human subjects involved in research. More information about taking part in a research study can be found on the Columbia University IRB website at: http://www.cumc.columbia.edu/dept/irb.

I have read the consent form and talked about this research study, including the purpose, procedures, risks, benefits and alternatives with the researcher. Any questions I had were answered to my satisfaction. I am aware that by signing below, I am agreeing to take part in this research study and that I can stop being in the study at any time. I am not waiving (giving up) any of my legal rights by signing this consent form. I will be given a copy of this consent form to keep for my records.

Signature

Study Subject

Print Name____________________Signature____________________Date__________

Person Obtaining Consent

Print Name____________________Signature____________________Date__________
