# Supplementary material for: “The Ultimate Decision Is Yours”: Exploring Patients’ Attitudes about the Overuse of Medical Interventions
Source: PLoS One. 2012 Dec 26;7(12):e52552. doi: 10.1371/journal.pone.0052552 (PMC3530438; doi:10.1371/journal.pone.0052552)
Supplement: Table S1 — Characteristics of focus group participants. (DOCX) [file pone.0052552.s004.docx]

Table S1: Characteristics of Focus Group Participants (n=43)

|  | Total Participants | Median Age | Women | Men | African-American | White | Hispanic | Race data not collected |
| --- | --- | --- | --- | --- | --- | --- | --- | --- |
| Atlanta A | 7 | 48 | 6 | 1 | 1 | 6 | 0 | ~~-~~ |
| Atlanta B | 8 | 50 | 5 | 3 | 1 | 7 | 0 | - |
| Chicago A | 10 | 50.5 | 6 | 4 | 2 | 8 | 0 | - |
| Chicago B | 8 | 52 | 3 | 5 | 2 | 5 | 1 | - |
| Tarrytown | 10 | 50 | 6 | 4 | ~~-~~ | ~~-~~ | ~~-~~ | 10 |
| Total | 43 | 50 | 26 | 17 | ~~-~~ | ~~-~~ | ~~-~~ | - |
